# Creating a Distinct Medication-Use System for Children at the Point of Care: The Time is Now

**DOI:** 10.3390/pharmacy3030072

**Published:** 2015-06-25

**Authors:** Richard Parrish, Ibolja Cernak

**Affiliations:** 1 Pharmacy Perioperative Care and Paediatrics, Regional Pharmacy Services—North Alberta Health Services, Royal Alexandra Hospital, 10240 Kingsway, NW, S103.5 ATC, Edmonton, AB T5H 3V9, Canada; 2 Military and Veterans’ Clinical Rehabilitation Research, University of Alberta, Edmonton, AB T6G 2G4, Canada

**Keywords:** AAP Committee on Drugs, medication-use system, off-label, pediatrics, pharmacotherapy

## Abstract

Children need a distinct medicines-use system designed explicitly for them since their continued inclusion in a system of prescription processing developed for adults generates insoluble risk points and workarounds. The American Academy of Pediatrics (AAP), in its policy statement released by the AAP Committee on Drugs in early 2014 about off-label use in children, posits that federal legislation on increased drug testing in children has been effective, as “there have been over 500 pediatric-specific labeling changes.” However, the AAP’s position has not changed materially since the original 2002 policy statement. Indeed, other health professionals, their organizations, or affiliated practice-based research network (PBRNs) mechanisms continue to be excluded from consideration, collaboration, or even honorable mention. It is noteworthy that most of the 500 labeling changes made since 1997 have addressed the scientific validity of indications for medication use in pediatric population without regard to pharmacotherapy formulation or monitoring. Medication use in children continues to be associated with an unacceptably high rate of adverse events, morbidity, and death. Children should no longer be “shoehorned” into the adult medication-use system, which faces challenges in addressing even the adult population’s needs. The time is now to design a multi-phasic, systematic approach to the pharmacotherapy of children. This paper will argue for the establishment of a distinct medication use system for children, a trans-disciplinary system designed thoughtfully and intentionally, not by convention, consensus, or imitation.

## 1. Introduction

In February 2014, the Committee on Drugs of the American Academy of Pediatrics (AAP) released a policy statement about the off-label drug use in children [1]. In the AAP’s statement, five complementary recommendations were generated covering their suggested use-system for children’s medications. The Committee defined “off-label” use as “that [which] is not included in the package insert (governmentally-approved labeling) for that drug [1]. They further define the role of the United States Food and Drug Administration (US-FDA) with regard to children as: (1) labeling arbiter; and (2) post-marketing monitor. Regarding therapeutic decision-making, the Committee reinforced the idea that off-label use is “neither experimentation or research…and does not warrant special consent or review” [1]. In summary, AAP posits that federal legislation to increase drug testing in children has been effective because “there have been over 500 pediatric-specific labeling changes” [1]. In essence, AAP’s updated policy statement on drugs for children has not changed materially since its original policy statement on the issue in 2002 [2]. It upholds the idea that child medication safety and use-systems are best secured and top-down through the relationship between the AAP and US-FDA, and with the exclusion of other health professionals, their organizations, or affiliated practice-based research (PBRNs) mechanisms. In this paper, we outline several issues not addressed in the AAP policy statement and illustrate how perpetuation of the current pharmacotherapy system used in pediatric population makes children needlessly vulnerable to drug-related morbidity and mortality. Further, we argue for the establishment of a separate medication-use system designed not by convention or imitation but intentionally for children.

## 2. Just How Safe is Medication Use in Children across the Continuum of Care?

A cursory review of the available literature reveals that medication-use in children has been associated with unacceptably high rate of adverse events, morbidity, and death. While some of these events may be due to the absence of validated labeling, a recent review on medication errors found that drug therapy problems occurring in non-inpatient settings are both underreported and lack consistent definitions [3]. It has been reported that computerized provider order entry systems (CPOE) in hospitals reduce the likelihood of certain errors in children by as much as 50% [4,5,6]. The few studies that identify and summarize pharmacotherapy problems and medication errors in children residing at home revealed an overall rate of 70.2 errors per 100 patients, and a median preventable adverse drug event (pADE) rate of 16.5% [7,8]. Zed and colleagues studied the natural course of pediatric emergency department (ED) visits, and found that medication-related ED visits and hospital admissions were both common and often preventable in pediatric patients, and resulted in significant healthcare resource utilization [9]. Regrettably, the current health care advisory services don’t have appreciable impact on the prevention of medication administration errors [10]; the lack of such influence is especially critical in the populations of those at the highest risk of these errors, such as the neonates and children receiving multiple medications [11,12]. Since 1995, the paucity and quality of research reports have allowed only a glimpse into the problems of pediatric drug therapy at the point-of-care. However, these studies reveal the tip of the iceberg related to the social and clinical burden of drug-related morbidity and mortality in children.

## 3. Off-Label Drug Use—What Is It?

What, then, is the meaning of drug use within the context of the term “off-label”? For pediatricians, the medical necessity in a particular patient indicates and justifies the drug use. However, a cursory examination of any product’s label after the US-FDA Modernization Act of 1997 illustrates that the “indication” is only one of the 20 label-components [13]. For example, changes to support a pediatric indication for palonosetron, a 5-hydroxytriptamine 3 inhibitor, as anti-emetic in children suffering from nausea and vomiting due to chemotherapy or after a surgical procedure, generated three label statement changes: (1) indication; (2) dosing; and (3) warning and precautions [14]. It is noteworthy that only the dosing information establishing a weight-based dosing regimen for children referred specifically to the pediatric population, whereas the indication and safety remarks remained generic without addressing the specificity of pediatric population. Moreover, since the US-FDA did not require the company to insert a labeling change that specified the methods of extemporaneous preparation of the palonosetron dosage, the company did not include such compounding information in its label change submission. In other words, while the US-FDA needed assurances from the company that a pharmacy could extemporaneously compound both intravenous and oral palonosetron doses reliably, it left the addition of the information on dose preparation to the product’s labeling optional. Hence, the company’s obligation was limited only to providing the data for Agency review, not necessarily to include population-specific information in the label change, as per their guidance below:

“You (the company) must also provide the following information for the compounded formulation (to US-FDA): Active ingredients, diluents, and suspending and sweetening agents; detailed step-by-step compounding instructions; packaging and storage requirements; and formulation stability information. You must provide instructions to pharmacists and health care providers for accurately measuring the volume of IV solution or oral formulation for compounding. Compounding must be performed at 3 or more pharmacies to demonstrate adequacy of the compounding method. Analytical data and statistical results on the compounding method must be provided to the Agency for review”.

[15]

Accordingly, inserting the fixed weight-based dosing statement into the label change information was sufficient. Nevertheless, while palonosetron now has a use indication and as such it is not an “off-label” drug, from a pediatrician’s perspective, there is no guidance on dose preparation for a pharmacist. Furthermore, a pharmacist would still consider the use of palonosetron in pediatric population “off-label”, since formulation of a child’s dose is not mentioned anywhere in its label. Importantly, to a nurse or a parent, the label doesn’t provide children-specific monitoring parameters for safety or effectiveness.

Taking together, it is clear that an “approved drug use” is a broader concept, and contains more components than the AAP supports. A significant labeling change should entail much more than establishing a use indication to justify medical necessity (and, therefore, third-party insurance reimbursement). Adding a label change related to indication without including the means for achievement—dose preparation and stability—is neither logical nor ethical. Other professionals have a similar and corresponding responsibility to the pediatric patient that is squarely within their scopes of practice, an appreciation of which is absent in AAP’s policy statement, US-FDA labeling requirements, and any company’s responsibility to public health. Interestingly, off-label use of any medicine in many EU countries requires the patient’s informed consent for the protection of a fundamental right of self-determination. Moreover, the need for informed consent should extend to other healthcare professionals involved because the need for an alternate (*i.e.*, compounded) product may be a viable option for the patient’s care [16]. Nevertheless, while providing informed consent may be a necessary step for mitigating drug-related morbidity and mortality, it is not sufficient when the system has no formalized follow-up mechanisms other than voluntary reporting.

## 4. Innovative Practice *vs.* Research—A False Dichotomy?

The following statement of the AAP’s Committee on Drugs is of essential importance: “Off-label use is neither incorrect nor investigational if based on sound scientific evidence, expert medical judgment, or published literature” [1]. Thus, off-label use could be construed as an innovative practice in many circumstances; as such, it is not particular to the practice of pediatrics. The APP’s policy statement would be much more useful if it would provide a clear definition of “innovative practice”, the means of achieving it, and the role of the Practice-Based Research Networks (PBRNs) within the scope of pragmatic research. Namely, discussing the role of the PBRNs in generating evidence-based data collection, objective analysis, and dissemination of knowledge would provide a lead to clinician-researchers and pharmacists interested in developing children-specific knowledge networks. Indeed, many professional groups, like the Children’s Oncology Group and the Pediatric Trials Network, are structured as PBRNs and aim to facilitate clinician-researcher engagement in treatment standardization and harmonization.

Our following example details the answer on the question: Innovative practice or research? Say that you are a pediatrician who prescribes fluconazole suspension for oral thrush at a dose of 6 mg/kg. You use fluconazole in your practice because a 2 mL dose of nystatin oral suspension no longer seems to be effective in these cases, although the mass-produced nystatin product has an established indication in children. You have treated all oral thrush cases with fluconazole in a similar manner for a number of years, and have discussed your approach with your colleagues. At a citywide journal club, you make an oral presentation summarizing the safety and benefits of your use-algorithm in the cases that you have treated. Then, according to the suggestion in the AAP policy statement, you write a case series for a publication in professional literature. Does this product selection and dosing approach constitute “innovative practice” or research?

The American College of Gynecology and Obstetrics (ACOG), another physician/colleague organization, has derived a very salient approach to this issue [17]. In making such a distinction, the following three questions should be answered: (1) to what degree does the innovation differ from standard practice; (2) what is the potential to harm the patient; and (3) what is the intent of the physician? In our example, using fluconazole varies from standard practice; it is potentially safer and more effective than nystatin. However, the pediatrician in question wanted not only to provide evidence for the need to change standard practice, but also to increase her chances for university tenure. Hence, the pediatrician’s practice could be considered research; as such, it should be subject to informed consent and peer review. Perhaps, a similar interrogatory could be applied to all forms of innovative practice in all health disciplines, including pediatrics and clinical pharmacy. Since the US-FDA or company marketing the oral suspension of fluconazole show no interest in developing children-specific knowledgebase, the PBRNs have great potential to address the types of above-listed pragmatic research questions and subsequently improve patient care in pediatric population.

Since the late 1990s, the Agency for Healthcare Quality and Research (AHRQ) through the Healthcare Research and Quality Act has supported the conceptual and actual local operation of PBRNs [18]. It is interesting to note AAP’s apparent oversight within the context of both the role of government AND medicine, in its efforts to facilitate generation of scientific evidence for supporting more responsive medication-use in children. The reasons for AAP not to mention PBRNs as a viable system for knowledge acquisition, synthesis and dissemination, are left to the reader to contemplate. Suffice it to say that the social and Congressional mandate of the US-FDA as the final arbiter of the facts about pharmaceuticals is incomplete without a systematic, comprehensive and singular focus on medication safety for children.

## 5. Children Need a Distinct Medication-Use System That Works for Them and Their Parents

From the moment when a provider evaluates a pediatric patient’s need for drug therapy, every potential choice might be linked to uncertainty and ambiguity. The prevalence of off-label use in children has been estimated at up to 80% depending on the definition of off-label, the jurisdiction, the setting, and the perspective related to the conducted study. Unfortunately, resources from governments, professional consortia, and corporations devoted to research and development have focused on the pharmacokinetics and pharmacodynamics of injectable drugs within very narrow segments of the pediatric population. Indeed, over the past 20 years, there were only sporadic scientific efforts to develop unique oral non-sterile dosage forms with a potential for mass production and standardization that would address the needs of pediatric patients with chronic diseases under the age of four years.

Accordingly, a multi-phasic, systematic approach to the pharmacotherapy of children is needed, which would: (1) integrate assessments for both safety and effectiveness at the point-of-care; (2) include information-sharing requirements along the continuum of care; and (3) request formulation standardization in order to optimize therapeutic outcomes. Such a system would incorporate organized and timely follow-up as well as feedback components in order to validate the expected outcomes. Furthermore, the point-of-care outcomes would be assessed by healthcare professionals with appropriate training and credentials, such as board-certified pediatric/clinical pharmacists. We envision a system, which would ascertain that medicines used in pediatric population are designed specifically for children; and their preparation and distribution is controlled and followed through independent clinical and prescription management structure. Such a system designed intentionally for children and not by default would support confidence between the patients and their families, pediatricians, pharmacists, and nurses at every stages of the healthcare.

We recommend creating two separate and distinct age-based medicines-use systems: One for those aged 17 and above, and the other, for those below the age of 17 years. Since the current system adopted by the US-FDA and AAP is aimed at validating only the indication for medicines-use, a new system is needed that focuses on children’s needs and addresses four additional areas of system design: (1) discovering, validating, and sharing new and enhanced methods for extemporaneous formulation/re-formulation; (2) enhancement of the infrastructure providing computer informatics that list scientifically-validated extemporaneous and mass produced products and allow bi-directional flow of complete information from prescription initiation to fulfillment; (3) establishment of collaborative practices between pediatricians and board certified pediatric clinical pharmacists to systematically assess, collect, and transmit outcomes information to a central repository using a pediatric minimum data set; and (4) pragmatic clinical trials through which teams of health professionals would have the opportunity to ask and answer important outcomes-based questions. To manage this child-specific medicines-use system, specially trained and certified clinical pharmacists need to be educated and consistently employed at the point-of-care in order to complement and enhance the pediatricians’ clinical work.

The need for fulfillment is at-hand. We need to avoid our historical blinders and gaps, and work together for the benefit of children. Re-packaging or re-positioning product lines or making bitter medicines more palatable for children to swallow only provides a false sense of security the current, one-size-fits-all system gives and will give to children and their parents. Ask any parent of a child with complex medical needs—the system does not work. We strongly advocate for children having their own distinct medicines-use system, which is designed with their needs at the forefront. Having children being included in a medication-use system developed for adults and modified using principles that are far from physiological and molecular features of characteristic for pediatric population makes children perpetually and needlessly vulnerable to medication errors at every step in their healthcare. The time to act is now.
